# Associations of cardiac stress biomarkers with incident type 2 diabetes and changes in glucose metabolism: KORA F4/FF4 study

**DOI:** 10.1186/s12933-020-01117-1

**Published:** 2020-10-16

**Authors:** Chaterina Sujana, Jochen Seissler, Jens Jordan, Wolfgang Rathmann, Wolfgang Koenig, Michael Roden, Ulrich Mansmann, Christian Herder, Annette Peters, Barbara Thorand, Cornelia Then

**Affiliations:** 1 Institute of Epidemiology, Helmholtz Zentrum München-German Research Centre for Environmental Health, Ingolstädter Landstrasse 1, 85764 Neuherberg, Germany; 2 Institute for Medical Information Processing, Biometry, and Epidemiology (IBE), Ludwig-Maximilians-Universität, Munich, Germany; 3 Pettenkofer School of Public Health, Munich, Germany; 4 Medizinische Klinik und Poliklinik IV, Klinikum der Universität München, Ludwig-Maximilians-Universität, Munich, Germany; 5 Institute of Aerospace Medicine, German Aerospace Center (DLR) and University of Cologne, Cologne, Germany; 6 Institute for Biometrics and Epidemiology, German Diabetes Center, Leibniz Center for Diabetes Research at Heinrich Heine University Düsseldorf, Düsseldorf, Germany; 7 German Center for Diabetes Research (DZD), Munich-Neuherberg, Germany; 8 Deutsches Herzzentrum München, Technische Universität München, Munich, Germany; 9 German Centre for Cardiovascular Research (DZHK), Partner Site Munich Heart Alliance, Munich, Germany; 10 Institute of Epidemiology and Medical Biometry, University of Ulm, Ulm, Germany; 11 Institute for Clinical Diabetology, German Diabetes Center, Leibniz Center for Diabetes Research at Heinrich Heine University Düsseldorf, Düsseldorf, Germany; 12 Division of Endocrinology and Diabetology, Medical Faculty, Heinrich Heine University Düsseldorf, Düsseldorf, Germany

**Keywords:** MR-proANP, Copeptin, CT-proET-1, MR-proADM, Cardiac stress biomarkers, Type 2 diabetes, Prediabetes, Insulin resistance, Incidence, Cohort study

## Abstract

**Background:**

High N-terminal pro-brain-type natriuretic peptide levels have been associated with a lower risk of type 2 diabetes mellitus (T2D). However, less is known about other cardiac stress biomarkers in this context. Here we evaluated the association of mid-regional pro-atrial natriuretic peptide (MR-proANP), C-terminal pro-arginine vasopressin (copeptin), C-terminal pro-endothelin-1 (CT-proET-1) and mid-regional pro-adrenomedullin (MR-proADM) with incident T2D and changes in glucose metabolism.

**Methods:**

We performed a prospective cohort study using data from the population-based KORA F4/FF4 study. 1773 participants (52.3% women) with MR-proANP measurements and 960 (52.7% women) with copeptin, CT-proET-1 and MR-proADM measurements were included. We examined associations of circulating plasma levels of MR-proANP, copeptin, CT-proET-1 and MR-proADM with incident T2D, the combined endpoint of incident prediabetes/T2D and with fasting and 2 h-glucose, fasting insulin, HOMA-IR, HOMA-B and HbA1c at follow-up. Logistic and linear regression models adjusted for age, sex, waist circumference, height, hypertension, total/HDL cholesterol ratio, triglycerides, smoking, physical activity and parental history of diabetes were used to compute effect estimates.

**Results:**

During a median follow-up time of 6.4 years (25th and 75th percentiles: 6.0 and 6.6, respectively), 119 out of the 1773 participants and 72 out of the 960 participants developed T2D. MR-proANP was inversely associated with incident T2D (odds ratio [95% confidence interval]: 0.75 [0.58; 0.96] per 1-SD increase of log MR-proANP). Copeptin was positively associated with incident prediabetes/T2D (1.29 [1.02; 1.63] per 1-SD increase of log copeptin). Elevated levels of CT-proET-1 were associated with increased HOMA-B at follow-up, while elevated MR-proADM levels were associated with increased fasting insulin, HOMA-IR and HOMA-B at follow-up. These associations were independent of previously described diabetes risk factors.

**Conclusions:**

High plasma concentrations of MR-proANP contributed to a lower risk of incident T2D, whereas high plasma concentrations of copeptin were associated with an increased risk of incident prediabetes/T2D. Furthermore, high plasma concentrations of CT-proET-1 and MR-proADM were associated with increased insulin resistance. Our study provides evidence that biomarkers implicated in cardiac stress are associated with incident T2D and changes in glucose metabolism.

## Background

Type 2 diabetes mellitus (T2D) is a known major risk factor for heart failure [1], but the identification of biological pathways linking both diseases remains challenging. In recent years, several circulating biomarkers implicated in cardiac stress conditions have been shown to be associated with diabetes risk factors. For example, N-terminal pro-B-type natriuretic peptide (NT-proBNP) and mid-regional pro-atrial natriuretic peptide (MR-proANP) were inversely associated with metabolic syndrome and insulin resistance [2–4]. Several novel cardiac stress biomarkers like C-terminal pro-arginine-vasopressin (copeptin), C-terminal pro-endothelin-1 (CT-proET-1) and mid-regional pro-adrenomedullin (MR-proADM) were positively associated with metabolic syndrome and insulin resistance in cross-sectional settings [5, 6]. Copeptin was also associated with a family history of T2D in a recent study [7]. Collectively, these findings implicate cardiac stress biomarkers in the pathogenesis of T2D.

To date, a growing number of studies suggest that low NT-proBNP levels are associated with incident T2D [8–10]. Similarly, low MR-proANP levels were associated with incident T2D [8, 11]. Conversely, copeptin, that is commonly known as a biomarker for diabetes insipidus [12, 13], was positively associated with incident T2D in some studies [14–16], but others observed this positive association only in women [17]. In comparison to NT-proBNP, MR-proANP and copeptin have been less widely investigated regarding their roles in the development of T2D.

Furthermore, cross-sectional studies have shown that levels of CT-proET-1 and MR-proADM were elevated in patients with T2D [5, 18], but the few existing prospective studies failed to provide evidence of their associations with incident T2D [8, 16]. However, several animal studies have demonstrated that high endothelin-1 but low adrenomedullin were involved in the development of insulin resistance [19, 20], suggesting that both vasoactive peptides may be associated with changes in glucose metabolism.

The present study aimed to evaluate plasma levels of MR-proANP, copeptin, CT-proET-1 and MR-proADM for their putative associations with incident T2D, the combined endpoint of incident prediabetes/T2D and traits of glycaemia and insulin resistance (fasting and 2 h-glucose, fasting insulin, homeostasis model assessment of insulin resistance (HOMA-IR) and β-cell function (HOMA-B) and haemoglobin A1c (HbA1c)) at follow-up.

## Methods

### Study design and participants

We performed a prospective cohort study using data from Cooperative Health Research in the Augsburg Region (KORA) F4 (2006–2008) and FF4 (2013–2014) studies. KORA F4 and FF4 are follow-up examinations of the fourth survey of the population-based KORA study (KORA S4; 1999–2001) conducted in the South of Germany. The study design has been described previously in detail [21]. The three examinations were carried out in accordance with Declaration of Helsinki, including obtaining written informed consent from all participants. The study was approved by the ethics board of the Bavarian Chamber of Physicians (Munich, Germany).

This study included all persons aged 32–81 years participating in both KORA F4 (as baseline in the present analysis) and KORA FF4 studies (follow-up). Baseline characteristics of KORA F4 participants who did not participate in KORA FF4 and thus were excluded from the current analysis are summarised in Additional file 1: Table S1. Reasons for non-participation were: individuals had died, moved out of the study area, refused, were too ill/not interested/too busy to participate, or could not be contacted. We further excluded participants with diabetes at baseline, unclear diabetes status at baseline and follow-up, self-reported history of myocardial infarction and stroke, non-fasting participants prior to oral glucose tolerance test (OGTT) and participants with missing values on cardiac stress biomarkers or covariables.

**Fig. 1:**
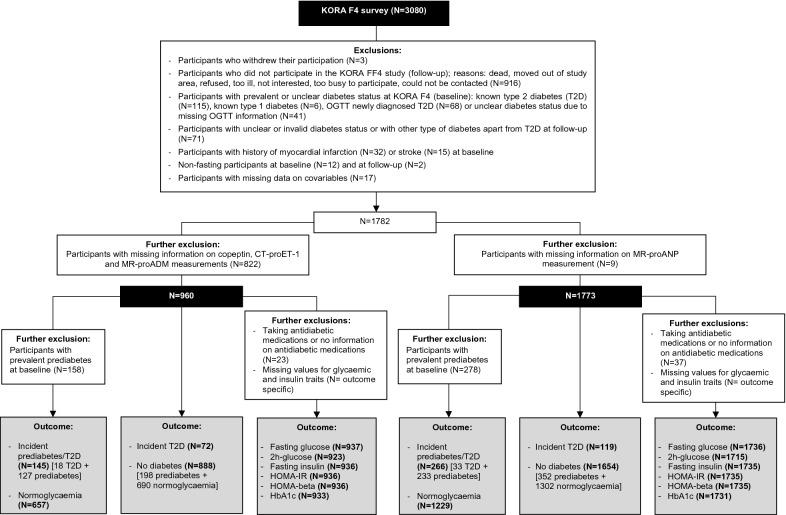
Flowchart showing sample sizes and reasons for exclusion

For analysing the association with incident T2D, 1773 nondiabetic participants at baseline (278 had prediabetes) with MR-proANP measurements and 960 (158 had prediabetes) with copeptin, CT-proET-1 and MR-proADM measurements were included. For analysing the association with incident prediabetes/T2D, 1495 normoglycaemic participants at baseline with MR-proANP measurements and 802 with copeptin, CT-proET-1 and MR-proADM measurements were included. For analysing the associations with traits of glycaemia and insulin resistance we excluded participants who were taking glucose-lowering medication at baseline and at follow-up. Exclusions are described in Fig. 1 in detail.

### Assessment of T2D and prediabetes

Known T2D was defined as self-reported diabetes that was validated through contacting the responsible physician or medical chart review or current self-reported use of glucose-lowering medication. Participants without known diabetes were assigned to receive a standard 75 g OGTT. Normoglycaemia was defined as having glucose after overnight fasting (fasting glucose) < 6.1 mmol/l and 2 hours after glucose solution intake (2 h-glucose) < 7.8 mmol/l; prediabetes as having fasting glucose ≥ 6.1 mmol/l but < 7.0 mmol/l and 2 h-glucose < 7.8 mmol/l (isolated impaired fasting glucose [IFG]) or fasting glucose < 6.1 mmol/l and 2 h-glucose ≥ 7.8 mmol/l but < 11.1 mmol/l (isolated impaired glucose tolerance [IGT]) or both IFG and IGT; newly diagnosed diabetes as having fasting glucose ≥ 7.0 mmol/l or 2 h-glucose ≥ 11.1 mmol/l [21, 22].

Incident T2D was defined as having normoglycaemia or prediabetes at baseline and known or newly diagnosed T2D at follow-up. Incident prediabetes/T2D was defined as having normoglycaemia at baseline and prediabetes or known or newly diagnosed T2D at follow-up.

### Laboratory measurements

During the baseline examinations in 2006–2008, blood samples from all participants were collected without stasis and kept at 4 °C until centrifugation. All included cardiac stress biomarkers were measured in plasma samples that were stored at − 80 °C until assayed. MR-proANP was measured in all KORA F4 study participants, while copeptin, CT-proET-1 and MR-proADM were measured in the first 1596 participants of the KORA F4 study. These biomarkers were assayed with novel sandwich fluoro-immunoassay (BRAHMS, Hennigsdorf, Berlin, Germany) using the automated system BRAHMS KRYPTOR. Copeptin, MR-proADM and CT-proET-1 were assayed simultaneously in 2010, while MR-proANP was assayed in 2016–2017. Intra- and inter-assay coefficients of variation were 3.5 and 3.4% for MR-proANP; 5.9 and 8.9% for copeptin; 4.8 and 6.9% for CT-proET-1; 4.5 and 7.8% for MR-proADM, respectively.

Glucose levels were measured in serum using a hexokinase method (GLUFlex, Dade Behring, Deerfield, USA) at baseline and an enzymatic colorimetric method on a Dimension Vista 1500 instrument (Siemens Healthcare Diagnostics Inc., Newark, NJ, USA) or the GLUC3 assay on a Cobas c702 instrument (Roche Diagnostics GmbH, Mannheim, Germany) at follow-up. Fasting insulin levels were measured in serum using an electrochemiluminescence immunoassay on a Cobas e602 instrument (Roche) at baseline and a solid-phase enzyme-labelled chemiluminescent immunometric assay on an Immulite 2000 systems analyser (Siemens) or an electrochemiluminescence immunoassay on a Cobas e602 instrument (Roche) at follow-up. HOMA-IR was calculated as (fasting insulin [µU/ml] x fasting glucose [mmol/l])/22.5 and HOMA-B was calculated as (fasting insulin [μU/ml] × 20)/(fasting glucose [mmol/l] − 3.5). HbA1c at baseline was assayed in haemolysed whole blood using high performance liquid chromatography on an Adams HA 8160 Haemoglobin Analysis System (Arkray Inc., distributed by A. Menarini Diagnostics, Florence, Italy) and at follow-up on Variant™ II Turbo HbA1c Kit–2.0 (Bio-Rad Laboratories Inc., Hercules, CA, USA). Calibration for different methods was performed as previously described [23].

Serum total cholesterol (TC) and high-density lipoprotein (HDL) were measured with enzymatic methods (CHOL Flex and AHDL Flex, Dade Behring, Marburg, Germany) and serum triglycerides with the GPO-PAP method (Dade Behring). All blood parameters, except for 2 h-glucose, were based on fasting blood samples.

### Assessment of other covariables

Trained medical interviewers collected information on medical history, lifestyle and parental history of diabetes of all participants as described elsewhere [24]. Smoking status was categorised into never, former or current smoking. Physical activity was assessed according to duration of leisure time sport activities with four possible answers: [1] > 2 h/week, [2] 1–2 h/week, [3] < 1 h/week, [4] none, separately in winter and in summer. A total score for physical activity was obtained by summing the possible answers in winter and in summer. Participants who had a total score ≥ 5 were classified as ‘physically inactive’, otherwise ‘physically active’. Parental history of diabetes was categorised into at least one parent with diabetes, no parent with diabetes or unknown (all remaining participants).

Waist circumference and height were measured by trained personnel as described previously [24]. Actual hypertension was defined as blood pressure ≥ 140/90 mmHg or use of antihypertensive medication given that the participants were aware of being hypertensive.

### Statistical analysis

Participants characteristics are reported as mean (standard deviation (SD)) or geometric mean (antilog of SD) for continuous variables and percentages for categorical variables stratified by cases vs non-cases of incident T2D and of incident prediabetes/T2D. Characteristics between cases and non-cases were compared using analysis of variance and Chi squared test for continuous and categorical variables, respectively. Characteristics were calculated for all included participants, i.e. 1773 participants for the analysis of incident T2D and 1495 for the analysis of incident prediabetes/T2D.

The associations of cardiac stress biomarkers with incident T2D and incident prediabetes/T2D were analysed by calculating odds ratio (OR) with 95% confidence interval (CI) in logistic regression models. The exact date of prediabetes and T2D manifestation during follow-up was unknown, thus we conducted logistic regression analysis rather than survival (time-to-event) analysis. Cardiac stress biomarkers were investigated as continuous measures per 1-SD increase. MR-proANP, copeptin and MR-proADM were log-transformed to approximate normality. The distribution of CT-proET-1 was approximately normal and it was thus not log-transformed. All included cardiac stress biomarkers were z-standardized.

We performed analysis of covariance to estimate the associations of cardiac stress biomarkers with traits of glycaemia and insulin resistance at follow-up. The effect estimates (beta) with 95% CI were computed by assigning follow-up values of the continuous traits as outcome variables and including the baseline values as covariables in the linear regression models. To approximate normality, the continuous trait variables were log-transformed and z-standardized.

All association analyses were adjusted for sex (male/female), age, waist circumference, and height (all continuous) (model 1), plus actual hypertension (yes/no), ratio of total/HDL cholesterol (TC/HDL) (continuous), triglyceride concentration (continuous), smoking (current/former/never), physical activity (inactive/active) and parental history of diabetes (at least one parent/unknown/no) (model 2). We also performed sex-stratified analyses and tested for interaction by sex.

Additionally, we calculated area under the receiver operating characteristic curve (AUC) and category-free net reclassification improvement (cfNRI) to quantify the added predictive value of the cardiac stress biomarkers beyond the conventional diabetes risk factors. Differences in AUC with 95% CI were computed using the method described by DeLong et al. [25]. Overall cfNRI represents the sum of net proportions of persons correctly assigned a higher predicted risk for cases (cfNRI_cases_) and a lower predicted risk for non-cases (cfNRI_non-cases_) [26]. The 95% CIs for cfNRIs were computed using the percentile bootstrap method with 1000 iterations.

All statistical analyses were performed in participants with complete data of baseline and follow-up measurements and were conducted with SAS version 9.4 (SAS Institute Inc., Cary, NC, USA), except for cfNRI calculation that was conducted with R version 3.6.3 [27]. The *P*-values presented were two-tailed, and *P* < 0.05 was considered statistically significant.

## Results

During a median follow-up time of 6.4 years (minimum: 5.1; 25th percentile: 6.0; 75th percentile: 6.6; maximum: 7.7), out of 1773 nondiabetic participants with MR-proANP measurements, 119 developed T2D, and out of 960 nondiabetic participants with copeptin, CT-proET-1 and MR-proADM measurements, 72 developed T2D. Furthermore, out of 1495 normoglycaemic participants with MR-proANP measurement, 266 developed prediabetes/T2D, and out of 802 participants with copeptin, CT-proET-1 and MR-proADM measurements, 145 developed prediabetes/T2D during follow-up.

The characteristics of study participants are presented in Table 1. The cases (both incident T2D and incident prediabetes/T2D) comprised more men than women. At baseline, the cases were on average older, had a higher waist circumference and TC/HDL ratio, higher triglyceride concentrations, were more frequently hypertensive, physically less active and more likely to have parents with diabetes. The cases had higher levels of MR-proANP, copeptin, CT-proET-1 and MR-proADM than the non-cases.

**Table 1:** Characteristics of study participants

	Incident T2D			Incident prediabetes/T2D		
	Cases^a^ N = 119	Non-cases^b^ N = 1654	*P*	Cases^c^ N = 266	Non-cases^d^ N = 1229	*P*
Male	56.3%	47.1%	0.052	54.5%	44.3%	*0.003*
Age, years	61.5 (10.8)	52.1 (11.8)	*< 0.001*	57.1 (11.3)	50.1 (11.4)	*< 0.001*
Waist circumference, cm	102.5 (13.0)	90.8 (13.3)	*< 0.001*	97.3 (11.7)	88.4 (12.2)	*< 0.001*
Height, cm	168.2 (9.4)	169.7 (9.5)	0.067	168.8 (9.5)	170.2 (9.6)	*0.032*
Actual hypertension	53.8%	26.5%	*< 0.001*	42.1%	20.3%	*< 0.001*
Parental diabetes history			*0.010*			*< 0.001*
At least 1 parent	30.3%	23.3%		29.3%	21.4%	
Unknown	21.0%	14.2%		21.8%	12.8%	
No diabetic parents	48.7%	62.5%		48.9%	65.8%	
Physically inactive	49.6%	39.3%	*0.027*	43.2%	37.2%	*0.034*
Smoking status			*0.011*			0.865
Current	10.1%	17.5%		18.1%	18.4%	
Former	34.5%	40.2%		36.8%	39.6%	
Never	55.5%	42.3%		45.1%	42.0%	
Ratio of total cholesterol/HDL	4.70 (1.21)	3.97 (1.14)	*< 0.001*	4.35 (1.20)	3.83 (1.09)	*< 0.001*
Triglycerides, mmol/l^e^	1.57 (1.68)	1.10 (1.71)	*< 0.001*	1.30 (1.71)	1.02 (1.67)	*< 0.001*
MR-proANP, pmol/l^e^	63.6 (1.7)	57.8 (1.6)	*0.026*	62.7 (1.6)	56.3 (1.6)	*< 0.001*
Copeptin, pmol/l^e,f^	6.29 (3.45)	5.19 (3.65)	0.225	7.22 (3.25)	4.93 (3.65)	*< 0.001*
CT-proET-1, pmol/l^f^	48.9 (9.6)	44.4 (11.6)	*< 0.001*	48.2 (13.6)	43.1 (10.9)	*< 0.001*
MR-proADM, nmol/l^e,f^	0.55 (1.24)	0.47 (1.26)	*< 0.001*	0.52 (1.27)	0.46 (1.25)	*< 0.001*

Characteristics were calculated in the cohort comprising of 1773 participants for the analysis of incident T2D and 1495 for the analysis of incident prediabetes/T2D. Data are presented as mean (SD) or geometric mean (antilog SD) for continuous variables and as percentages for categorical variables

Italic values indicate significant *P* values (*P* < 0.05)

CT-proET-1, C-terminal pro-endothelin-1; MR-proADM, mid-regional pro-adrenomedullin; MR-proANP, mid-regional pro-atrial natriuretic peptide; SD, standard deviation; T2D, type 2 diabetes

^a^ No diabetes (normoglycaemia or prediabetes) at baseline and T2D at follow-up

^b^ No diabetes (normoglycaemia or prediabetes) at baseline and follow-up

^c^ Normoglycaemia at baseline and prediabetes or T2D at follow-up

^d^ Normoglycaemia at baseline and follow-up

^e^ Data with skewed distributions are presented as geometric mean (antilog SD)

^f^ Data were calculated in 960 nondiabetic participants at baseline (72 developed T2D and 888 remained nondiabetic at follow-up) and 802 normoglycaemic participants (145 developed prediabetes/T2D and 657 remained normoglycaemic at follow-up)

Differences were also observed for traits of glycaemia and insulin resistance between cases and non-cases. The cases displayed higher levels of fasting and 2 h-glucose, fasting insulin, HOMA-IR, HOMA-B and HbA1c than the non-cases at baseline and at follow-up (Table 2).

**Table 2:** Traits of glycaemia and insulin resistance at baseline and follow-up

	Incident T2D			Incident prediabetes/T2D		
	Cases^a^	Non-cases^b^	*P*	Cases^c^	Non-cases^d^	*P*
Measurements at baseline						
Fasting serum glucose, mmol/l^e,f^	5.78 (1.09)	5.12 (1.10)	*< 0.001*	5.38 (1.07)	5.01 (1.08)	*< 0.001*
2 h serum glucose, mmol/l^e,g^	7.89 (1.23)	5.51 (1.29)	*< 0.001*	6.04 (1.20)	5.12 (1.25)	*< 0.001*
Fasting serum insulin, µU/ml^e,h^	13.46 (1.66)	8.14 (1.63)	*< 0.001*	9.98 (1.64)	7.46 (1.57)	*< 0.001*
HOMA-IR^e,h^	3.46 (1.71)	1.85 (1.70)	*< 0.001*	2.39 (1.68)	1.66 (1.62)	*< 0.001*
HOMA-B^e,h^	119.9 (1.7)	103.8 (1.6)	*0.004*	107.6 (1.6)	101.9 (1.5)	0.073
HbA1c, mmol/mol^i^	39.1 (3.5)	35.1 (3.5)	*< 0.001*	36.9 (3.2)	34.4 (3.3)	*< 0.001*
Measurements at follow-up						
Fasting serum glucose, mmol/l^e,f^	6.82 (1.22)	5.35 (1.10)	*< 0.001*	5.93 (1.12)	5.20 (1.08)	*< 0.001*
2h serum glucose, mmol/l^e,g^	11.75 (1.31)	5.73 (1.31)	*< 0.001*	8.02 (1.25)	5.19 (1.24)	*< 0.001*
Fasting serum insulin, µU/ml^e,h^	15.18 (1.69)	8.65 (1.73)	*< 0.001*	12.62 (1.72)	7.71 (1.66)	*< 0.001*
HOMA-IR^e,h^	4.60 (1.87)	2.06 (1.82)	*< 0.001*	3.33 (1.79)	1.78 (1.71)	*< 0.001*
HOMA-B^e,h^	95.0 (1.7)	96.0 (1.7)	0.864	106.2 (1.7)	92.5 (1.6)	*< 0.001*
HbA1c, mmol/mol^i^	43.2 (9.3)	35.2 (3.8)	*< 0.001*	37.8 (4.8)	34.6 (3.5)	*< 0.001*

Characteristics were calculated in the sample for analysing the association of cardiac stress biomarkers with glycaemic and insulin traits at the follow-up after excluding participants taking glucose-lowering medication. Data are presented as mean (SD) or geometric mean (antilog SD)

Italic values indicate significant *P* values (*P* < 0.05)

HbA1c, haemoglobin A1c; HOMA-B, homeostasis model assessment of beta-cell function; HOMA-IR, homeostasis model assessment of insulin resistance; SD, standard deviation; T2D, type 2 diabetes

^a^ No diabetes (normoglycaemia or prediabetes) at baseline and T2D at follow-up

^b^ No diabetes (normoglycaemia or prediabetes) at baseline and follow-up

^c^ Normoglycaemia at baseline and prediabetes or T2D at follow-up

^d^ Normoglycaemia at baseline and follow-up

^e^ Data with skewed distributions are presented as geometric mean (antilog SD)

^f^ Data were calculated in 1736 nondiabetic participants at baseline (84 developed T2D and 1652 remained nondiabetic) and 1484 normoglycaemic participants (257 developed prediabetes/T2D and 1227 remained normoglycaemic at follow-up)

^g^ Data were calculated in 1715 nondiabetic participants at baseline (63 developed T2D and 1652 remained nondiabetic) and 1478 normoglycaemic participants (251 developed prediabetes/T2D and 1227 remained normoglycaemic at follow-up)

^h^ Data were calculated in 1735 nondiabetic participants at baseline (84 developed T2D and 1651 remained nondiabetic) and 1484 normoglycaemic participants (257 developed prediabetes/T2D and 1227 remained normoglycaemic at follow-up)

^i^ Data were calculated in 1731 nondiabetic participants at baseline (84 developed T2D and 1647 remained nondiabetic) and 1479 normoglycaemic participants (256 developed prediabetes/T2D and 1223 remained normoglycaemic at follow-up)

### Associations between cardiac stress biomarkers and incident T2D

MR-proANP was inversely associated with incident T2D in model 1. The OR [95% CI] was 0.70 [0.55; 0.89], *P *= 0.004 per 1-SD increase of log MR-proANP. The association was attenuated, but remained significant after additional adjustment according to model 2 (OR [95% CI] 0.75 [0.58; 0.96], *P *= 0.025; Table 3). When we excluded participants with prediabetes at baseline the associations remained significant (OR [95% CI] 0.60 [0.37; 0.96], *P *= 0.033; Additional file 1: Table S2).

**Table 3:** Associations between cardiac stress biomarkers and incident type 2 diabetes

Biomarkers	N_cases/non-cases_	Model	OR [95% CI]	*P*
MR-proANP	119/1645	Model 1	*0.70* [*0.55; 0.89*]	*0.004*
		Model 2	*0.75* [*0.58; 0.96*]	*0.025*
Copeptin	72/888	Model 1	1.03 [0.78; 1.36]	0.824
		Model 2	1.05 [0.79; 1.40]	0.730
CT-proET-1	72/888	Model 1	0.93 [0.69; 1.25]	0.626
		Model 2	0.82 [0.59; 1.14]	0.234
MR-proADM	72/888	Model 1	0.87 [0.62; 1.20]	0.389
		Model 2	0.85 [0.59; 1.21]	0.358

ORs [95% CI] were calculated per 1-SD increment of cardiac stress biomarkers

Model 1: adjusted for age, sex, waist circumference and height

Model 2: Model 1 + actual hypertension, ratio of total cholesterol and HDL, triglycerides, smoking status, physical activity and parental history of diabetes

Italic values indicate significant *P* values (*P* < 0.05)

CI, confidence interval; CT-proET-1, C-terminal pro-endothelin-1; MR-proADM, mid-regional pro-adrenomedullin; MR-proANP, mid-regional pro-atrial natriuretic peptide; OR, odds ratio; SD, standard deviation

Copeptin, CT-proET-1 and MR-proADM were not significantly associated with incident T2D (Table 3). Sex-specific associations between the cardiac stress biomarkers and incident T2D are presented in Additional file 1: Table S3.

The AUC [95% CI] of conventional T2D risk factors (model 2) without any cardiac stress biomarkers predicting incident T2D in the full study population was 0.833 [0.799; 0.867] and 0.853 [0.814; 0.891] in the subpopulation with copeptin, CT-proET-1 and MR-proADM measurements. None of the cardiac stress biomarkers individually improved the AUC significantly on top of model 2 (Additional file 1: Table S4). The overall cfNRI was significantly improved when MR-proANP was added to model 2 (cfNRI_overall_ [95% CI] 0.211 [0.015; 0.466]), but cfNRI for cases and non-cases were not significantly improved (cfNRI_cases_ [95% CI] 0.109 [− 0.018; 0.273] and cfNRI_non-cases_ [95% CI] 0.102 [− 0.009; 0.231]). None of the other cardiac stress biomarkers significantly improved the cfNRI when added to model 2 (Additional file 1: Table S4).

### Associations between cardiac stress biomarkers and incident prediabetes/T2D

Copeptin was positively associated with incident prediabetes/T2D. The OR [95% CI] was 1.30 [1.03; 1.63], *P *= 0.027 per 1-SD increase of log copeptin in model 1. The association remained similar after further adjustment according to model 2 (OR [95% CI] 1.29 [1.02; 1.63], *P *= 0.033; Table 4). In a sensitivity analysis excluding participants who progressed from normoglycaemia at baseline to T2D at follow-up, copeptin was also positively associated with incident prediabetes alone (OR [95% CI] 1.43 [1.10; 1.86], *P *= 0.008; Additional file 1: Table S2).

**Table 4:** Associations between cardiac stress biomarkers and the combined incident prediabetes/type 2 diabetes endpoint

Biomarkers	N_cases/non-cases_	Model	OR [95% CI]	*P*
MR-proANP	266/1229	Model 1	0.91 [0.76; 1.09]	0.297
		Model 2	0.94 [0.78; 1.14]	0.539
Copeptin	145/657	Model 1	*1.30* [*1.03; 1.64*]	*0.027*
		Model 2	*1.29* [*1.02; 1.63*]	*0.033*
CT-proET-1	145/657	Model 1	1.15 [0.95; 1.40]	0.154
		Model 2	1.15 [0.94; 1.41]	0.169
MR-proADM	145/657	Model 1	1.09 [0.85; 1.38]	0.497
		Model 2	1.08 [0.84; 1.39]	0.543

ORs [95% CI] were calculated per 1-SD increment of cardiac stress biomarkers

Model 1: adjusted for age, sex, waist circumference and height

Model 2: Model 1 + actual hypertension, ratio of total cholesterol and HDL, triglycerides, smoking status, physical activity and parental history of diabetes

Italic values indicate significant *P* values (*P* < 0.05)

CI, confidence interval; CT-proET-1, C-terminal pro-endothelin-1; MR-proADM, mid-regional pro-adrenomedullin; MR-proANP, mid-regional pro-atrial natriuretic peptide; OR, odds ratio; SD, standard deviation

We observed no significant associations of MR-proANP, CT-proET-1 and MR-proADM with incident prediabetes/T2D (Table 4). Sex-specific associations between the cardiac stress biomarkers and incident prediabetes/T2D are presented in Additional file 1: Table S5.

The AUC [95% CI] of the conventional T2D risk factors (model 2) predicting incident prediabetes/T2D without any cardiac stress biomarkers was 0.779 [0.750; 0.807] in the total study population and 0.796 [0.760; 0.832] in the subpopulation with available copeptin, CT-proET-1 and MR-proADM measurements. The addition of the cardiac stress biomarkers individually to model 2 did not significantly improve the AUC and overall cfNRI (Additional file 1: Table S6).

### Associations of cardiac stress biomarkers with traits of glycaemia and insulin resistance at follow-up

**Fig. 2:**
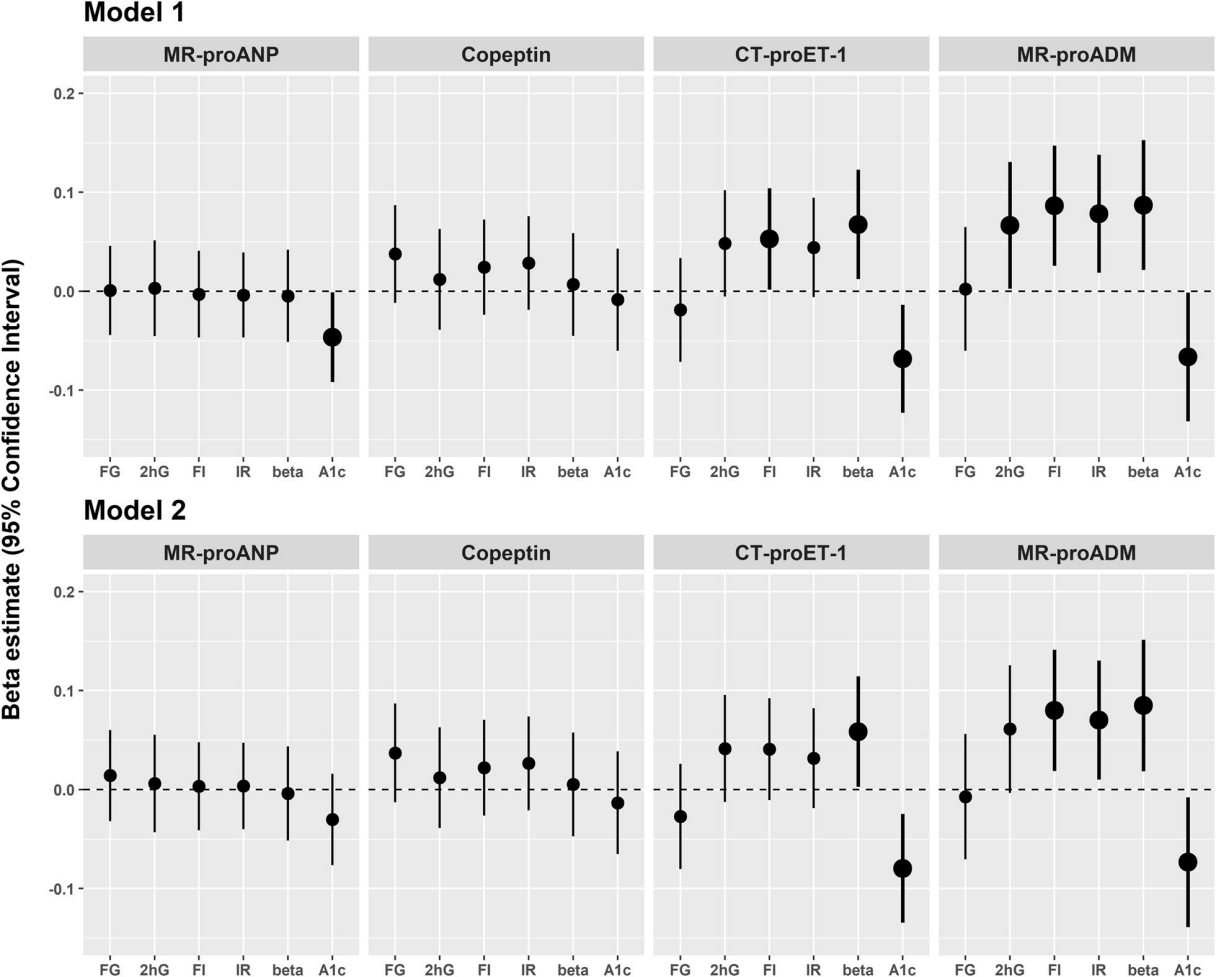
Associations of cardiac stress biomarkers with continuous traits of glycaemia and insulin resistance at follow-up. Thick plots show significant associations. The analyses were adjusted for baseline value of glycaemic or insulin traits, age, sex, waist circumference and height (model 1) and were further adjusted for actual hypertension, ratio of total cholesterol and HDL, triglycerides, smoking status, physical activity and parental history of diabetes (model 2). Abbreviations: 2 hG, 2 h-glucose; A1c, haemoglobin A1c; beta, homeostasis model assessment of beta-cell function; CT-proET-1, C-terminal pro-endothelin-1; FG, fasting glucose; FI, fasting insulin; IR, homeostasis model assessment of insulin resistance; MR-proADM, mid-regional pro-adrenomedullin; MR-proANP, mid-regional pro-atrial natriuretic peptide

Elevated MR-proANP levels were only significantly associated with reduced HbA1c in model 1, but the association was attenuated to non-significance after further adjustment in model 2 (Fig. 2). Copeptin was not significantly associated with any of the assessed continuous traits at follow-up.

Elevated levels of CT-proET-1 and MR-proADM at baseline were significantly associated with higher fasting insulin and HOMA-B at follow-up in model 1. Elevated MR-proADM levels were also significantly associated with higher 2 h-glucose and HOMA-IR in model 1 (Fig. 2, model 1). Further adjustment (model 2) attenuated the positive association between CT-proET-1 and fasting insulin and also the positive association between MR-proADM and 2 h-glucose to non-significance (Fig. 2, model 2). Additionally, we observed significant inverse associations of CT-proET-1 and MR-proADM with HbA1c, even after further adjustment in model 2.

## Discussion

In the current study elevated MR-proANP levels were associated with a lower risk of incident T2D, elevated copeptin levels were associated with a higher risk of incident prediabetes/T2D, and elevated CT-proET-1 and MR-proADM levels were associated with increases in several traits of insulin resistance during the follow-up period. Overall, the examined cardiac stress biomarkers did not substantially improve the prediction of incident T2D and incident prediabetes/T2D when added to the models containing conventional diabetes risk factors. As previous research demonstrated, the predictive ability of established diabetes risk scores is rarely considerably improved by the addition of single novel biomarkers, however, this does not preclude relevant associations between the biomarkers and T2D [28].

### MR-proANP and incident T2D

Our results describing the inverse association of MR-proANP with incident T2D are consistent with results from previous studies [8, 11]. Additionally, we observed a significant improvement of overall cfNRI but not AUC in predicting incident T2D after the addition of MR-proANP to model 2. This may be explained by the fact that the cfNRI is more sensitive to small changes in predicted risk after the addition of a new marker than the AUC [29]. We are not aware of any other studies addressing improvement in T2D prediction by the addition of MR-proANP.

Furthermore, our study adds evidence that higher MR-proANP levels were associated with decreased HbA1c at follow-up in a model adjusted for age, sex, waist circumference and height. However, further adjustment attenuated this association to non-significance.

Low atrial natriuretic peptide (ANP) levels were associated with the activation of the renin-angiotensin system [30], which in turn could promote the development of insulin resistance and thus T2D [31]. However, in the present study we observed no significant associations of baseline MR-proANP with any of the assessed insulin traits at follow-up. By contrast, a recent investigation from the MDC study [32] among 2243 nondiabetic participants who were followed up for 16.5 years reported a significant inverse association of MR-proANP with fasting insulin and HOMA-IR at follow-up. However, the authors reported no significant associations of MR-proANP with fasting and 2 h-glucose at follow-up as seen in our results.

Findings from experimental studies suggest that natriuretic peptides directly affect metabolism in skeletal muscle and adipose tissue. Natriuretic peptides were shown to induce fat oxidative capacity, reduce lipotoxicity and enhance insulin signalling in skeletal muscle and promote lipolysis, browning and glucose uptake in adipose tissue [33]. ANP also inhibited the secretion of adipokines and cytokines involved in inflammation and insulin resistance [34]. These biological effects of ANP improved insulin sensitivity and blood glucose control [33] and may thereby explain the inverse association of MR-proANP and incident T2D.

### Copeptin and incident prediabetes/T2D

Our findings on the positive association between copeptin and incident prediabetes/T2D among normoglycaemic participants at baseline are in accordance with findings from the DESIR study [15] showing a positive association between copeptin and incident IFG/T2D among participants with normal fasting glucose at baseline. However, in the present study, copeptin was not significantly associated with incident T2D among nondiabetic participants at baseline. Similar to our finding, the FINRISK study [8] also observed no significant associations between copeptin and incident T2D among nondiabetic participants at baseline. In contrast, investigations from the MDC study [14] and the British Regional Heart Study [35] reported positive associations between copeptin and incident T2D among nondiabetic participants at baseline. These associations were stronger in participants without IFG than in all nondiabetic participants at baseline. Recently, higher copeptin levels were reported in participants with prediabetes than in participants with T2D [7]. This finding corroborates our results on a more pronounced association of copeptin with incident prediabetes alone than with the combined incident prediabetes/T2D. Unfortunately, our study is underpowered to examine the association between copeptin and incident T2D alone among participants with normoglycaemia at baseline.

Previously, elevated copeptin was reported to be associated with increased insulin resistance [14, 15] which may partly explain our findings. Of note, we also observed a trend towards a positive association of copeptin with fasting insulin and HOMA-IR. The active peptide arginine vasopressin directly stimulates glucagon and insulin release from pancreas and induces hepatic glycogenolysis [36]. Interestingly, higher rather than lower copeptin levels were reported in participants treated with empagliflozin, which is known to reduce hyperglycaemia, than in participants treated with placebo [37]. Although this finding might be due to a mild volume depletion caused by sodium-glucose cotransporter-2 inhibition of empagliflozin [37], further studies are needed to understand the mechanism of copeptin-induced hyperglycaemia.

The utility of copeptin in the prediction of prediabetes/T2D is not widely understood. In the present analysis, we did not observe a significantly improved prediction of incident prediabetes/T2D after the addition of copeptin to models containing conventional diabetes risk factors. However, Abbasi et al. [17] shows that copeptin significantly improved the prediction of T2D in addition to conventional diabetes risk factors in women, but not in men. More studies are needed to understand the clinical significance of copeptin in the prediction of prediabetes/T2D beyond the known role of predicting clinical outcomes of heart failure [38] and other major cardiovascular events in T2D patients [39].

### CT-proET-1, MR-proADM and insulin resistance

In the present study we also observed positive associations of CT-proET-1 and MR-proADM with increased traits of insulin resistance during follow-up in nondiabetic participants. Our findings extend findings from previous cross-sectional analyses demonstrating that CT-proET-1 and MR-proADM were positively associated with metabolic risk factors and insulin resistance [5, 40, 41]. To the best of our knowledge, this is the first study to examine the prospective associations of CT-proET-1 and MR-proADM with fasting insulin, HOMA-IR and HOMA-B in a nondiabetic population.

Several in vitro and experimental studies have shown that endothelin-1 stimulated insulin secretion directly from pancreas [19, 42]. Endothelin-1 also limited insulin action [43] and decreased adiponectin mRNA levels in adipocytes [44]. These biological effects may lead to the development of insulin resistance and thus, support our findings. The positive association between CT-proET-1 with higher HOMA-B at follow-up in our study was most likely a consequence of its positive association with insulin resistance. Furthermore, there is evidence that endothelin-1 inhibits glucose uptake in human skeletal muscle [45] that may further explain the trend of a positive association between CT-proET-1 and 2 h-glucose at follow-up in our study as 2 h-glucose mainly reflects muscle glucose uptake [46]. Interestingly, we also observed an inverse association between CT-proET-1 and HbA1c. The reason for this finding is still unknown and further confirmation is needed.

Regarding the association between MR-proADM and insulin resistance, we found that higher MR-proADM levels were positively rather than inversely associated with fasting insulin, HOMA-IR and HOMA-B at follow-up. Our findings are contradictory to the results of a previous animal study [20]. The mechanistic evidence linking MR-proADM and insulin resistance is not well understood and controversial. In the previous animal study [20] adrenomedullin deficiency increased oxidative stress and induced insulin resistance. Adrenomedullin also inhibited insulin secretion on the pancreatic islets and reduced insulin levels accompanied by an increase in postprandial glucose [47]. In contrast, high adrenomedullin levels stimulated interleukin-6 and remarkably potentiated stimulatory effects of tumor necrosis factor-α, interleukin-1β and lipopolysaccharide that contribute to the development of insulin resistance [48]. A recent epidemiological study further demonstrated that high MR-proADM was associated with increased BMI and waist circumference at follow-up [49]. The study also reported that high MR-proADM was associated with lower fasting glucose levels that contradicts evidence from the animal models [47]. More studies are needed to confirm our findings on the associations between high MR-proADM and increased insulin resistance at follow-up.

## Strengths and limitations

Strengths of the current study include the prospective study design, the population-based sample and the availability of OGTT data at baseline and follow-up. This enabled us to examine prospective associations between the included cardiac stress biomarkers with progression from normoglycaemia to prediabetes/T2D and with the continuous traits of glycaemia and insulin resistance at follow-up.

Limitations of this study are: Our study has a relatively low number of incident T2D cases and is therefore not sufficiently powered to examine associations with incident T2D among participants with normoglycaemia and prediabetes at baseline separately. Furthermore, participants who participated in KORA FF4 tended to be healthier than those who did not participate, which could have introduced some selection bias. Due to the lack of data on history of heart failure at baseline, we were unable to exclude participants with heart failure. Due to the lack of follow-up data on the included cardiac stress biomarkers, we were also unable to examine changes of the biomarkers in the progression towards diabetes. Our study participants were predominantly European descent, which means that further studies need to confirm our findings in other ethnic groups.

## Conclusions

High plasma concentrations of MR-proANP were associated with a lower risk of incident T2D, whereas high plasma concentrations of copeptin were associated with an increased risk of incident prediabetes/T2D. Furthermore, high plasma concentrations of CT-proET-1 and MR-proADM were associated with increased insulin resistance during follow-up time. Our study provides evidence that biomarkers implicated in cardiac stress are associated with incident T2D and changes in glucose metabolism.

## Supplementary information

**Additional file 1: Table S1.** Characteristics of KORA F4 study participants stratified by participation in the follow-up study KORA FF4. **Table S2.** Associations of cardiac stress biomarkers with incident type 2 diabetes and incident prediabetes among different subgroups. **Table S3.** Sex-specific associations between cardiac stress biomarkers and incident type 2 diabetes. **Table S4.** Predictive performance of cardiac stress biomarkers in predicting incident type 2 diabetes. **Table S5.** Sex-specific association between cardiac stress biomarkers and incident prediabetes/type 2 diabetes. **Table S6.** Predictive performance of cardiac stress biomarkers in predicting incident prediabetes/type 2 diabetes.

## Data Availability

The data are subject to national data protection laws and restrictions were imposed by the Ethics Committee of the Bavarian Chamber of Physicians to ensure data privacy of the study participants. Therefore, data cannot be made freely available in a public repository. However, data can be requested through an individual project agreement with KORA via the online portal KORA.passt (https://epi.helmholtz-muenchen.de/).

## Abbreviations

ANP: Atrial natriuretic peptide
AUC: Area under the receiver operating characteristic curve
cfNRI_cases_: Category-free net reclassification improvement for cases
cfNRI_non-cases_: Category-free net reclassification improvement for non-cases
cfNRI_overall_: Overall category-free net reclassification improvement
CI: Confidence interval
CT-proET-1: C-terminal pro-endothelin-1
HbA1c: Haemoglobin A1c
HDL: High density lipoprotein
HOMA-B: Homeostasis model assessment of insulin resistance β-cell function
HOMA-IR: Homeostasis model assessment of insulin resistance
IFG: Isolated impaired fasting glucose
IGT: Isolated impaired glucose tolerance
KORA: Cooperative Health Research in the Region of Augsburg
MR-proADM: Mid-regional pro-adrenomedullin
MR-proANP: Mid-regional pro-atrial natriuretic peptide
NT-proBNP: N-terminal pro-B-type natriuretic peptide
OGTT: Oral glucose tolerance test
OR: Odds ratio
SD: Standard deviation
TC: Total cholesterol
T2D: Type 2 diabetes mellitus
