# Supplementary material for: Associations of cardiac stress biomarkers with incident type 2 diabetes and changes in glucose metabolism: KORA F4/FF4 study
Source: Cardiovasc Diabetol. 2020 Oct 16;19:178. doi: 10.1186/s12933-020-01117-1 (PMC7566143; doi:10.1186/s12933-020-01117-1)
Supplement: Supplementary file 1 — Additional file 1: Table S1. Characteristics of KORA F4 study participants stratified by participation in the follow-up study KORA FF4. Table S2. Associations of cardiac stress biomarkers with incident type 2 diabetes and incident prediabetes among different subgroups. Table S3. Sex-specific associations between cardiac stress biomarkers and incident type 2 diabetes. Table S4. Predictive performance of cardiac stress biomarkers in predicting incident type 2 diabetes. Table S5. Sex-specific association between cardiac stress biomarkers and incident prediabetes/type 2 diabetes. Table S6. Predictive performance of cardiac stress biomarkers in predicting incident prediabetes/type 2 diabetes. [file 12933_2020_1117_MOESM1_ESM.docx]

**Additional File 1** to manuscript ‘Associations of cardiac stress biomarkers with incident type 2 diabetes and changes in glucose metabolism: KORA F4/FF4 study’ (**Sujana et al.,** 2020)

**Table S1. Characteristics of KORA F4 study participants stratified by participation in the follow-up study KORA FF4**

|  | **Participation in KORA FF4 study** | | | **P** |
| --- | --- | --- | --- | --- |
|  | **Yes**  **N=2161** | **No** | |  |
|  |  | **Due to death**  **N=169** | **Other reasons ^a^**  **N=747** |  |
| Male | 48.6% | 64.5% | 43.6% | <0.001 |
| Age in years, *mean (SD)* | 54.0 (12.4) | 70.9 (9.3) | 58.7 (14.0) | <0.001 |
| Waist in cm, *mean (SD) ^b^* | 92.9 (14.0) | 101.1 (13.6) | 95.1 (13.7) | <0.001 |
| Height in cm, *mean (SD) ^c^* | 169.5 (9.5) | 166.2 (8.9) | 167.0 (9.77) | <0.001 |
| Actual hypertension | 33.1% | 67.9% | 46.8% | <0.001 |
| Parental history of diabetes |  |  |  | 0.005 |
| At least 1 parent | 24.8% | 27.2% | 21.7% |  |
| Unknown | 15.5% | 26.6% | 18.9% |  |
| None | 59.6% | 46.2% | 59.4% |  |
| Physically inactive | 42.0% | 61.5% | 52.5% | <0.001 |
| Smoking status |  |  |  | 0.056 |
| Current | 16.8% | 15.7% | 21.3% |  |
| Former | 41.4% | 50.6% | 36.1% |  |
| Never | 41.9% | 33.7% | 42.6% |  |
| Ratio of total cholesterol/HDL, *mean (SD) ^d^* | 4.04 (1.18) | 4.09 (1.14) | 4.18 (1.19) | 0.016 |
| Triglycerides in mmol/l, *geometric mean (antilog SD) ^d^* | 1.17 (1.75) | 1.30 (1.69) | 1.30 (1.72) | <0.001 |
| MR-proANP in pmol/l, *geometric mean (antilog SD) ^e^* | 59.9 (1.61) | 116.5 (1.93) | 69.6 (1.73) | <0.001 |
| Copeptin in pmol/l, *geometric mean (antilog SD) ^f^* | 5.48 (3.63) | 10.26 (2.92) | 6.00 (4.01) | <0.001 |
| MR-proADM in nmol/l, *geometric mean (antilog SD) ^f^* | 0.50 (1.29) | 0.73 (1.37) | 0.55 (1.34) | <0.001 |
| CT-proET-1 in pmol/l, *mean (SD) ^f^* | 46.2 (12.7) | 66.5 (22.1) | 49.8 (13.4) | <0.001 |
| Diabetes status at baseline |  |  |  | <0.001 |
| Normoglycemia | 75.0% | 36.7% | 62.2% |  |
| Prediabetes | 14.4% | 24.9% | 19.4% |  |
| T2D | 8.5% | 33.7% | 13.7% |  |
| T1D | 0.3% | 0.0% | 0.3% |  |
| Medication induced diabetes | 0.0% | 0.6% | 0.0% |  |
| Unclear diabetes status | 1.9% | 4.1% | 4.4% |  |
| HbA1c in mmol/mol, *mean (SD) ^d^* | 36.6 (5.9) | 41.1 (8.8) | 38.2 (8.1) | <0.001 |
| Fasting serum glucose in mmol/l, *geometric mean (antilog SD) ^g^* | 5.31 (1.16) | 5.90 (1.24) | 5.46 (1.20) | <0.001 |
| 2h serum glucose in mmol/l, *geometric mean (antilog SD) ^h^* | 5.80 (1.36) | 6.91 (1.41) | 6.16 (1.39) | <0.001 |
| Fasting serum insulin in microU/ml, *geometric mean (antilog SD) ^i^* | 8.91 (1.76) | 11.8 (1.90) | 9.84 (1.77) | <0.001 |
| HOMA-IR, *geometric mean (antilog SD) ^j^* | 2.04 (1.83) | 2.68 (2.00) | 2.25 (1.84) | <0.001 |
| HOMA-B, *geometric mean (antilog SD) ^j^* | 105.3 (1.60) | 113.9 (1.85) | 108.3 (1.66) | 0.124 |

^a^ other reasons: individuals moved out of the study area, refused to participate, were too ill/not interested/too busy to participate or could not be contacted.

^b^ mean (SD) was calculated in 2155, 166 and 742 participants who participated and did not participate KORA FF4 due to death and other reasons, respectively.

^c^ mean (SD) was calculated in 2158, 166 and 744 participants who participated and did not participate KORA FF4 due to death and other reasons, respectively.

^d^ mean (SD) or geometric mean (antilog SD) was calculated in 2159, 167 and 746 participants who participated and did not participate KORA FF4 due to death and other reasons, respectively.

^e^ geometric mean (antilog SD) was calculated in 2137, 167 and 731 participants who participated and did not participate KORA FF4 due to death and other reasons, respectively.

^f^ mean (SD) or geometric mean (antilog SD) was calculated in 1171, 84 and 340 participants who participated and did not participate KORA FF4 due to death and other reasons, respectively.

^g^ geometric mean (antilog SD) was calculated in 2146, 158 and 735 participants who participated and did not participate KORA FF4 due to death and other reasons, respectively.

^h^ geometric mean (antilog SD) was calculated in 1999, 117 and 644 participants who participated and did not participate KORA FF4 due to death and other reasons, respectively.

^i^ geometric mean (antilog SD) was calculated in 2126, 157 and 726 participants who participated and did not participate KORA FF4 due to death and other reasons, respectively.

^j^ geometric mean (antilog SD) was calculated in 2039,125 and 674 participants who participated and did not participate KORA FF4 due to death and other reasons, respectively.

**Table S2. Associations of cardiac stress biomarkers with incident type 2 diabetes and incident prediabetes among different subgroups**

| Biomarkers | Model | OR [95%CI] | | |
| --- | --- | --- | --- | --- |
|  |  | Normoglycemia to T2D | Normoglycemia to Prediabetes | Prediabetes to T2D |
| MR-proANP | *N_cases/non-cases_* | *33 / 1229* | *233 / 1229* | *86 / 192* |
|  | Model 1 | **0.57 [0.36; 0.90]; P=0.015** | 0.97 [0.80; 1.16]; P=0.721 | 1.04 [0.77; 1.41]; P=0.796 |
|  | Model 2 | **0.60 [0.37; 0.96]; P=0.033** | 1.00 [0.83; 1.21]; P=0.987 | 1.07 [0.77; 1.48]; P=0.705 |
| Copeptin | *N_cases/non-cases_* | *18 / 657* | *127 / 657* | *54 / 104* |
|  | Model 1 | 0.80 [0.51; 1.25]; P=0.323 | **1.44 [1.11; 1.87]; P=0.006** | 1.45 [0.98; 2.12]; P=0.060 |
|  | Model 2 | 0.80 [0.50; 1.27]; P=0.341 | **1.43 [1.10; 1.86]; P=0.008** | **1.56 [1.04; 2.34]; P=0.033** |
| CT-proET-1 | *N_cases/non-cases_* | *18 / 657* | *127 / 657* | *54 / 104* |
|  | Model 1 | 0.88 [0.50; 1.56]; P=0.671 | 1.18 [0.97; 1.43]; P=0.098 | 1.18 [0.78; 1.80]; P=0.433 |
|  | Model 2 | 0.88 [0.49; 1.60]; P=0.678 | 1.18 [0.96; 1.45]; P=0.116 | 0.95 [0.59; 1.55]; P=0.845 |
| MR-proADM | *N_cases/non-cases_* | *18 / 657* | *127 / 657* | *54 / 104* |
|  | Model 1 | 0.72 [0.39; 1.31]; P=0.281 | 1.15 [0.89; 1.47]; P=0.287 | 0.99 [0.63; 1.55]; P=0.967 |
|  | Model 2 | 0.72 [0.38; 1.35]; P=0.305 | 1.13 [0.88; 1.46]; P=0.345 | 0.93 [0.57; 1.52]; P=0.774 |

ORs [95%CI] were calculated per 1-SD increment of cardiac stress biomarkers.

Model 1: adjusted for age, sex, waist circumference and height.

Model 2: Model 1 + actual hypertension, ratio of total cholesterol and HDL, triglyceride, smoking status, physical activity, parental history of diabetes.

Abbreviations: CI, confidence interval; CT-proET-1, C-terminal pro-endothelin-1; MR-proADM, mid-regional pro-adrenomedullin; MR-proANP, mid-regional pro-atrial natriuretic peptide; OR, odds ratio; SD, standard deviation; T2D, type 2 diabetes.

**Table S3. Sex-specific associations between cardiac stress biomarkers and incident type 2 diabetes**

| Biomarkers | Model | P-values for  sex interaction | Sex-specific OR [95%CI] | |
| --- | --- | --- | --- | --- |
|  |  |  | Women | Men |
| MR-proANP | *N_cases/non-cases_* |  | *52 / 875* | *67 / 779* |
|  | Model 1 | 0.408 | 0.71 [0.48; 1.03]; P=0.072 | **0.70 [0.51; 0.97]; P=0.030** |
|  | Model 2 | 0.843 | 0.73 [0.49; 1.09]; P=0.125 | 0.77 [0.55; 1.08]; P=0.129 |
| Copeptin | *N_cases/non-cases_* |  | *29 / 477* | *43 / 411* |
|  | Model 1 | 0.053 | 0.80 [0.56; 1.14]; P=0.220 | 1.48 [0.92; 2.40]; P=0.107 |
|  | Model 2 | **0.042** | 0.79 [0.53; 1.18]; P=0.251 | 1.54 [0.93; 2.55]; P=0.094 |
| CT-proET-1 | *N_cases/non-cases_* |  | *29 / 477* | *43 / 411* |
|  | Model 1 | 0.670 | 0.96 [0.58; 1.59]; P=0.863 | 0.90 [0.62; 1.30]; P=0.578 |
|  | Model 2 | 0.627 | 0.93 [0.55; 1.56]; P=0.771 | 0.75 [0.49; 1.16]; P=0.197 |
| MR-proADM | *N_cases/non-cases_* |  | *29 / 477* | *43 / 411* |
|  | Model 1 | 0.149 | 1.17 [0.71; 1.93]; P=0.532 | 0.70 [0.46; 1.08]; P=0.106 |
|  | Model 2 | 0.246 | 1.24 [0.72; 2.13]; P=0.439 | 0.65 [0.40; 1.04]; P=0.071 |

ORs [95%CI] were calculated per 1-SD increment of cardiac stress biomarkers.

Model 1: adjusted for age, waist circumference and height.

Model 2: Model 1 + actual hypertension, ratio of total cholesterol and HDL, triglycerides, smoking status, physical activity and parental history of diabetes.

Abbreviations: CI, confidence interval; CT-proET-1, C-terminal pro-endothelin-1; MR-proADM, mid-regional pro-adrenomedullin; MR-proANP, mid-regional pro-atrial natriuretic peptide; OR, odds ratio; SD, standard deviation.

**Table S4. Predictive performance of cardiac stress biomarkers in predicting incident type 2 diabetes**

| Biomarkers | Model | AUC [95%CI] | ΔAUC [95%CI] | cfNRI_overall_ [95%CI] | cfNRI_cases_ [95%CI] | cfNRI_non-cases_ [95%CI] |
| --- | --- | --- | --- | --- | --- | --- |
| **N=1773** | | | | | | |
| None | Model 1 | 0.798 [0.761; 0.835] | - | - | - | - |
|  | Model 2 | 0.833 [0.799; 0.867] | - | - | - | - |
| MR-proANP | Model 1 | 0.803 [0.767; 0.840] | 0.005 [-0.006; 0.016] | **0.378 [0.168; 0.611]** | **0.176 [0.041; 0.339]** | **0.202 [0.099; 0.290]** |
|  | Model 2 | 0.836 [0.804; 0.869] | 0.003 [-0.004; 0.011] | **0.211 [0.015; 0.466]** | 0.109 [-0.018; 0.273] | 0.102 [-0.009; 0.231] |
| **N= 960** | | | | | | |
| None | Model 1 | 0.813 [0.770; 0.856] | - | - | - | - |
|  | Model 2 | 0.853 [0.814; 0.891] | - | - | - | - |
| Copeptin | Model 1 | 0.813 [0.769; 0.856] | 0.000 [-0.002; 0.001] | -0.070 [-0.142; 0.314] | 0.417 [-0.493; 0.592] | -0.486 [-0.521; 0.568] |
|  | Model 2 | 0.852 [0.814; 0.890] | -0.001 [-0.003; 0.001] | -0.105 [-0.167; 0.364] | 0.361 [-0.441; 0.571] | -0.466 [-0.475; 0.535] |
| CT-proET-1 | Model 1 | 0.814 [0.771; 0.857] | 0.001 [0.000; 0.002] | 0.080 [-0.118; 0.346] | 0.111 [-0.173; 0.273] | -0.032 [-0.064; 0.184] |
|  | Model 2 | 0.852 [0.813; 0.890] | -0.001 [-0.003; 0.002] | 0.040 [-0.124; 0.392] | -0.111 [-0.206; 0.276] | 0.151 [-0.132; 0.316] |
| MR-proADM | Model 1 | 0.812 [0.767; 0.856] | -0.001 [-0.007; 0.004] | -0.024 [-0.161; 0.332] | -0.056 [-0.194; 0.195] | 0.032 [-0.047; 0.227] |
|  | Model 2 | 0.851 [0.812; 0.889] | -0.002 [-0.006; 0.003] | 0.015 [-0.176; 0.399] | -0.028 [-0.183; 0.234] | 0.043 [-0.079; 0.245] |

Model 1: adjusted for age, sex, waist circumference and height.

Model 2: Model 1 + actual hypertension, ratio of total cholesterol and HDL, triglycerides, smoking status, physical activity and parental history of diabetes.

Abbreviations: AUC, area under the receiver operating characteristic curve; cfNRI_cases_: category-free net reclassification improvement for cases; cfNRI_non-cases_: category-free net reclassification improvement for non-cases; cfNRI_overall_: overall category-free net reclassification improvement; CI, confidence interval; CT-proET-1, C-terminal pro-endothelin-1; MR-proADM, mid-regional pro-adrenomedullin; MR-proANP, mid-regional pro-atrial natriuretic peptide; ΔAUC, difference in AUC between models with and without cardiac stress biomarkers.

**Table S5. Sex-specific association between cardiac stress biomarkers and incident prediabetes/type 2 diabetes**

| Biomarkers | Model | P-values for  sex interaction | Sex-specific OR [95%CI] | |
| --- | --- | --- | --- | --- |
|  |  |  | Women | Men |
| MR-proANP | *N_cases/non-cases_* |  | *107 / 684* | *126 / 545* |
|  | Model 1 | 0.893 | 0.92 [0.70; 1.21]; P=0.547 | 1.01 [0.78; 1.30]; P=0.929 |
|  | Model 2 | 0.507 | 0.93 [0.70; 1.23]; P=0.599 | 1.07 [0.82; 1.39]; P=0.637 |
| Copeptin | *N_cases/non-cases_* |  | *56 / 372* | *71 / 285* |
|  | Model 1 | 0.155 | 1.24 [0.91; 1.70]; P=0.178 | **1.87 [1.15; 3.02]; P=0.011** |
|  | Model 2 | 0.216 | 1.16 [0.84; 1.62]; P=0.363 | **1.82 [1.12; 2.94]; P=0.015** |
| CT-proET-1 | *N_cases/non-cases_* |  | *56 / 372* | *71 / 285* |
|  | Model 1 | **0.032** | **1.33 [1.05; 1.69]; P=0.018** | 0.99 [0.72; 1.35]; P=0.931 |
|  | Model 2 | **0.042** | **1.36 [1.03; 1.78]; P=0.029** | 0.97 [0.69; 1.36]; P=0.866 |
| MR-proADM | *N_cases/non-cases_* |  | *56 / 372* | *71 / 285* |
|  | Model 1 | **0.003** | **1.54 [1.08; 2.19]; P=0.018** | 0.85 [0.60; 1.21]; P=0.358 |
|  | Model 2 | **0.007** | 1.45 [0.98; 2.14]; P=0.063 | 0.86 [0.59; 1.25]; P=0.428 |

The ORs [95%CI] were calculated per 1-SD increment of cardiac stress biomarkers.

Model 1: adjusted for age, waist circumference and height.

Model 2: Model 1 + actual hypertension, ratio of total cholesterol and HDL, triglycerides, smoking status, physical activity and parental history of diabetes.

Abbreviations: CI, confidence interval; CT-proET-1, C-terminal pro-endothelin-1; MR-proADM, mid-regional pro-adrenomedullin; MR-proANP, mid-regional pro-atrial natriuretic peptide; OR, odds ratio; SD, standard deviation.

**Table S6. Predictive performance of cardiac stress biomarkers in predicting incident prediabetes/type 2 diabetes**

| Biomarkers | Model | AUC [95%CI] | ΔAUC [95%CI] | cfNRI_overall_ [95%CI] | cfNRI_cases_ [95%CI] | cfNRI_non-cases_ [95%CI] |
| --- | --- | --- | --- | --- | --- | --- |
| **N= 1495** | | | | | | |
| None | Model 1 | 0.758 [0.729; 0.788] | - | - | - | - |
|  | Model 2 | 0.779 [0.750; 0.807] | - | - | - | - |
| MR-proANP | Model 1 | 0.758 [0.728; 0.788] | 0.000 [-0.003; 0.003] | 0.127 [-0.091; 0.280] | 0.083 [-0.095; 0.171] | 0.045 [0.024; 0.138] |
|  | Model 2 | 0.778 [0.750; 0.807] | -0.001 [-0.002; 0.001] | 0.057 [-0.081; 0.202] | 0.068 [-0.109; 0.170] | -0.011 [-0.046; 0.087] |
| **N= 802** | | | | | | |
| None | Model 1 | 0.768 [0.729; 0.807] | - | - | - | - |
|  | Model 2 | 0.796 [0.760; 0.832] | - | - | - | - |
| Copeptin | Model 1 | 0.775 [0.737; 0.813] | 0.007 [-0.004; 0.018] | 0.111 [-0.036; 0.305] | **0.517 [0.364; 0.624]** | **-0.406 [-0.482; -0.250]** |
|  | Model 2 | 0.802 [0.767; 0.837] | 0.006 [-0.003; 0.015] | 0.072 [-0.108; 0.280] | **0.448 [0.229; 0.550]** | **-0.376 [-0.437; -0.163]** |
| CT-proET-1 | Model 1 | 0.776 [0.738; 0.814] | 0.008 [0.000; 0.015] | 0.045 [-0.133; 0.250] | 0.007 [-0.112; 0.148] | 0.038 [-0.050; 0.147] |
|  | Model 2 | 0.802 [0.766; 0.837] | 0.006 [-0.001; 0.012] | 0.031 [-0.116; 0.308] | 0.021 [-0.103; 0.193] | 0.011 [-0.060; 0.146] |
| MR-proADM | Model 1 | 0.771 [0.732; 0.809] | 0.003 [-0.001; 0.006] | 0.109 [-0.097; 0.317] | 0.062 [-0.108; 0.187] | 0.047 [-0.033; 0.171] |
|  | Model 2 | 0.798 [0.762; 0.833] | 0.002 [-0.001; 0.004] | 0.097 [-0.098; 0.312] | 0.034 [-0.101; 0.181] | 0.062 [-0.039; 0.159] |

Model 1: adjusted for age, sex, waist circumference and height.

Model 2: Model 1 + actual hypertension, ratio of total cholesterol and HDL, triglycerides, smoking status, physical activity and parental history of diabetes.

Abbreviations: AUC, area under the receiver operating characteristic curve; cfNRI_cases_: category-free net reclassification improvement for cases; cfNRI_non-cases_: category-free net reclassification improvement for non-cases; cfNRI_overall_: overall category-free net reclassification improvement; CI, confidence interval; CT-proET-1, C-terminal pro-endothelin-1; MR-proADM, mid-regional pro-adrenomedullin; MR-proANP, mid-regional pro-atrial natriuretic peptide; ΔAUC, difference in AUC between models with and without cardiac stress biomarkers.
